# Regenerated Cellulose Filaments from the Recycling of Textile Cotton Waste Using Ionic Liquids

**DOI:** 10.1021/acsomega.5c01867

**Published:** 2025-05-07

**Authors:** Beatriz Barbosa de Brito, Beatriz Krüger, Lucas Souza da Silva, Cintia Marangoni, Andrea Cristiane Krause Bierhalz

**Affiliations:** † Department of Textile Engineering, 28117 Federal University of Santa Catarina, UFSC, João Pessoa 2750, Blumenau 89036-256, Santa Catarina, Brazil; ‡ Department of Chemical Engineering and Food Engineering, Federal University of Santa Catarina, UFSC, Eng. Agronômico Andrei Cristian Ferreira s/n, Florianópolis 88040-900, Santa Catarina, Brazil

## Abstract

In this study, cellulose filaments were obtained from the dissolution of an industrial cotton residue in the ionic liquids (ILs) 1-ethyl-3-methylimidazolium chloride ([Emim]­Cl) and 1-ethyl-3-methylimidazolium acetate ([Emim]­OAc) using dimethyl sulfoxide (DMSO) as the cosolvent. Cellulose regeneration was carried out in water or ethanol coagulation baths, and the effect of IL and coagulant on the degree of polymerization (DP), morphology, crystallinity, and thermal and mechanical properties was evaluated. Both ILs promoted the complete dissolution of cellulose, with the process in [Emim]­OAc occurring faster and with less fiber swelling. The obtained filaments exhibited a homogeneous appearance and a dense morphology, and it was noted that the optical brightener present in the cotton residue was maintained in the filament composition. The dissolution and regeneration processes promoted the depolymerization of cellulose, with significant differences between the ILs and the coagulant. The highest depolymerization was observed for the filaments resulting from the dissolution in [Emim]­Cl. X-ray diffraction analysis indicated a change in the crystalline structure of cellulose I of the residue to an amorphous structure in the filaments, except for the filament from dissolution in [Emim]­OAc and coagulation in ethanol, which presented a type II cellulose structure. Thermal stability was reduced for all filaments, with the lowest degradation temperature observed for the filament from dissolution in [Emim]Cl and coagulation in ethanol. This filament also obtained inferior mechanical properties as a result of low DP and crystallinity. The elastic modulus of the other filaments (10–13 GPa) was similar to that of regenerated fibers such as viscose and modal. Among the IL-coagulant systems evaluated, [Emim]­OAc-ethanol resulted in the most promising mechanical, thermal, and morphological properties.

## Graphical abstract

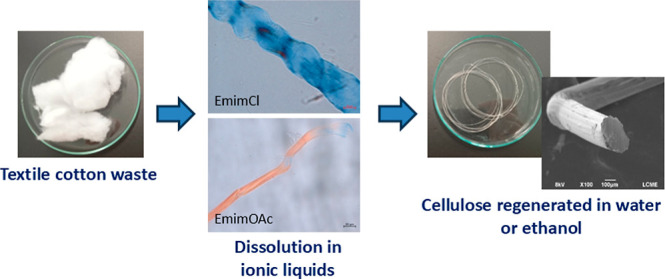

## 1 Introduction

Textile production has increased worldwide, driven by population growth, improved living standards, and fast fashion trends.^1^ Cotton is the most used natural fiber in the textile industry, known for its softness, breathability, and absorbency.^2^ However, this fiber has negative impacts throughout its lifecycle. For instance, traditional cotton cultivation involves high water and energy consumption and pesticide use.^3^ The spinning and weaving processes are associated with energy consumption, CO_2_ emissions, and the generation of various solid wastes (such as fiber lint, yarn waste, off-spec fabrics, fabric scraps, etc.). Additionally, postconsumer textiles are mostly landfilled, impacting biodiversity, water pollution, and greenhouse gas emissions.^4,5^

This scenario has made the recycling of cotton waste an emerging concern.^6^ Cotton is composed mainly of cellulose. Therefore, obtaining regenerated cellulose fibers from cotton waste represents an alternative to improve sustainability in the textile sector. However, regenerated fiber production also poses environmental challenges, attributed to the impossibility of melting cellulose and its inherent difficulty in dissolving. The strong intra- and intermolecular hydrogen bonds with highly ordered crystalline domains of cellulose result in low solubility in common solvents.^7,8^

Regenerated cellulose can be obtained by derivatizing and nonderivatizing processes. In derivatizing processes, such as the Viscose process, the chemical structure of the starting cellulose is modified, forming an intermediate compound, which is further dissolved and regenerated. Although commercially successful, derivatizing processes have been associated with serious environmental burdens.^9^ Therefore, efforts have been made to develop solvent systems capable of directly dissolving cellulose, thereby enabling greener processes.

In nonderivatizing processes, an organic solvent directly dissolves cellulose by disrupting intra- and intermolecular interactions between cellulose chains, with no intermediate compound formation.^10,11^ Several nonderivatizing solvent systems have been used to dissolve cellulose, such as cuprammonium, LiCl/DMAc, *N*-methylmorpholine-*N*-oxide (NMMO), and ionic liquids (ILs).

ILs, defined as pure molten salts composed of pairs of organic ions and metal counterions, have demonstrated excellent capacity for solubilizing cellulose with the potential for solvent recovery.^12,13^ Imidazolium ILs have also stood out among the various possibilities due to their high dissolution capacity. Their hydrophilic character facilitates the disruption of cellulose’s intermolecular hydrogen bond, weakening its hydrophobic interactions and enabling the production of new products.^14,15^ In these ILs, cations and anions play synergistic roles in cellulose dissolution. Studies involving different imidazolium ILs showed that those with acetate and chloride anions tend to exhibit good dissolution properties.^16^

Despite the effectiveness of cellulose dissolution in ILs, the dissolution rate in pure ILs is slow, partly due to the high viscosities of the solutions. The high viscosity also impacts spinning, limiting large-scale production. However, the dissolution efficiency of cellulose in ILs can be improved by molecular cosolvents, such as dimethyl sulfoxide (DMSO).^17,18^ The presence of DMSO is believed to improve the solvation capacity of ILs by lowering the system’s viscosity, thereby facilitating mass transport.^19^

After dissolution, regenerated cellulose fibers are formed in a coagulation bath comprising aprotic solvents. This process involves simultaneous diffusion of the IL into the coagulation bath and diffusion of the coagulant into the cellulose solution. The formation process and, consequently, the characteristics of regenerated cellulose fibers are influenced by the interactions between the IL and the coagulant.^13^

In this study, regenerated cellulose filaments were produced from the dissolution of textile cotton waste in imidazolium-based ILs and DMSO. The influence of the type of IL and the use of water or ethanol as a coagulating agent on the filaments’ mechanical, thermal, crystalline, and morphological properties was evaluated. Despite the extensive literature on cellulose dissolution in ILs, research on their impact on the properties of these regenerated materials using textile waste as a cellulose source is still limited.

## 2 Materials and Methods

### 2.1 Materials

Optically brightened white cotton waste from the textile brushing process, donated by a company from Brazil, was used as the cellulose raw material. ILs 1-ethyl-3-methylimidazolium chloride ([Emim]­Cl) and 1-ethyl-3-methylimidazolium acetate ([Emim]­OAc) (Sigma-Aldrich, USA) were used as solvents for cellulose dissolution. Other reagents, including bis­(ethylenediamine)­copper­(II) hydroxide solution (CUEN) 1 M (Sigma-Aldrich, USA), ethanol 96%, and dimethyl sulfoxide (DMSO), were all of analytical grade.

### 2.2 Cellulose Dissolution and Filament Production

Cellulose dissolution was performed according to Ferreira Knihs et al.^20^ Samples of cotton waste (0.09 g), previously dried, were added to 3 g of ILs at 110 °C in a glycerin bath under magnetic stirring at 400 rpm. The process was evaluated by polarized light optical microscopy, and the dissolution was considered complete when the solution was microscopically homogeneous without undissolved fibrils. Based on this criterion, the dissolution time was 60 min for [Emim]­OAc and 90 min for [Emim]­Cl.

After this period, 1.5 g of cosolvent DMSO was added to the IL/cellulose system, keeping the stirring for 10 min. The resulting solution was immediately transferred to a 10 mL syringe connected to a hose with an internal diameter of 3.15 mm. The set syringe/hose was horizontally fixed on an infusion pump (Fresenius Kabi, model Injectomat Agilia) operating at a spinning rate of 45 mL/h. The cellulose-IL-DMSO solution was extruded into a coagulation bath containing deionized water or ethanol at room temperature (∼25 °C) for filament solidification, followed by manual drawing. The filament was washed several times with deionized water to remove the residual IL. Finally, the filament was dried in an oven at 60 °C for 30 min. The presence of the optical brightener in the formed filaments was evaluated in a Colorcheker Executive light cabinet (Adexim Comexim, Brazil) using UV light.

### 2.3 Characterization

#### 2.3.1 Degree of Polymerization

The average DP of the cotton waste and regenerated cellulose fibers was calculated using the intrinsic viscosity method of ASTM D1795-13R2021.^21^ The cellulose sample (0.018 g) was immersed in a flask with 35 mL of a 0.5 M CUEN solution and sphere glasses. The mixture was stirred in an orbital shaker (NL-343-01, New Lab) at 150 rpm for 3 h at 25 °C under a nitrogen atmosphere. After this period, 7 mL of the solution were inserted into a viscosimeter Canon-Fenske n° 75, which was maintained in a water bath at 25 °C. The flow time was then recorded, and the DP was determined by eq 1.

1 DP=[η]×190

In eq 1, [η] is the intrinsic viscosity obtained by plotting log­[(ηrel-1)/*c*] against c and extrapolating the straight line through the points to *c* = 0. Relative viscosity [ηrel] is obtained by the ratio of outflow times of the CUEN-cellulose solution (*t*) and the outflow time of the CUEN solution (*t*_0_).

#### 2.3.2 Scanning Electron Microscopy

Scanning Electron Microscopy (SEM) images of the regenerated fibers were recorded with a JEOL M-6390LV scanning electron microscope (Japan) at an accelerating voltage of 8 kV. The samples were sputtered with gold (Leica, model EM SCD 500, Germany) before observation to enhance conductivity.

#### 2.3.3 X-ray Diffraction Analysis

X-ray measurements were conducted on a Miniflex600 Rigaku X-ray diffractometer (Japan) operated at 40 kV and 30 mA. Samples were scanned in the 2θ range of 5–90°. Regenerated cellulose filaments were ground before analysis. The crystallinity index (CrI) was calculated by Segal’s method^22^ using eq 2

2 $$CrI=\frac{I_{200}-I_{am}}{I_{200}}\times 100$$

where *I*_200_ is the height intensity of the crystal plane (at 22.6° of 2θ for cellulose *I*), and the *I*_am_ refers to the minimum intensity of diffraction attributed to amorphous cellulose between planar reflections (110)/(200) for cellulose *I*.

#### 2.3.4 Fourier Transform Infrared Spectroscopy

Fourier transform infrared spectroscopy (FTIR) spectra of the cotton waste and regenerated cellulose filaments were recorded in attenuated total reflectance mode using a Cary 660 FTIR spectrophotometer (Agilent Technologies, USA) from 4000 to 500 cm^–1^ with a resolution of 4 cm^–1^, and 32 average scans.

#### 2.3.5 Thermogravimetric Analysis

Thermal analysis of the cotton waste and filaments was conducted using an STA 449-F3 Jupiter (NETZSCH, Germany). Samples (10 mg) were heated at 10 °C/min from 25 to 600 °C under a nitrogen atmosphere with a flow rate of 60 mL/min.

#### 2.3.6 Mechanical Properties

Tensile strength (TS), tenacity, elongation at break, and Young’s modulus of the regenerated filaments were evaluated according to the ASTM D2256/D2256 M standard.^23^ The tests were performed using a texturometer (TA.XT Plus, Stable Micro Systems, UK) at a crosshead speed of 2 mm/min and an initial grip spacing of 10 cm. At least 10 replicates were analyzed for each filament type. The filament thickness of samples with a 15 cm length was measured by a micrometer (0.001 mm, Digimess, Brazil) at 5 different positions.

The tenacity of the filaments is obtained by the ratio between the force at break and the linear density. The linear density (*T*), expressed in Tex, was determined by the ratio between the mass of the filament (*g*) and the length of the filament (km).

### 2.4 Statistical Analysis

DP and mechanical properties of the filaments were presented as the mean with standard deviation. Statistical analysis was performed by one-way analysis of variance, followed by Tukey’s test to detect differences of means at *p* < 0.05 using the Statistica 13.5 software.

## 3 Results and Discussion

The dissolution of the cotton residue in the two ILs was monitored by polarized light microscopy. Some images at different dissolution times are shown in Figure 1. Practically no undissolved fibers were observed after 60 min of processing with [Emim]­OAc. In contrast, [Emim]Cl still showed some swollen fibers at 60 min, with complete dissolution occurring at 90 min (image not shown). The slower dissolution of [Emim]Cl compared to [Emim]­OAc has already been reported by Elhi et al.^16^ and may be attributed to the reconnection of the cellulose chains via chloride hydrogen bond bridges after the initial disruption of hydrogen bonds. Therefore, these new hydrogen bonds must be broken again to separate the chains.^24^ This secondary bond formation does not occur with acetate anions due to the presence of the hydrophobic methyl group. Li et al.^25^ also mention that the H-bonds formed by Cl^–^ cannot effectively separate the cellulose chain, thereby contributing to slower dissolution.

**Figure 1:**
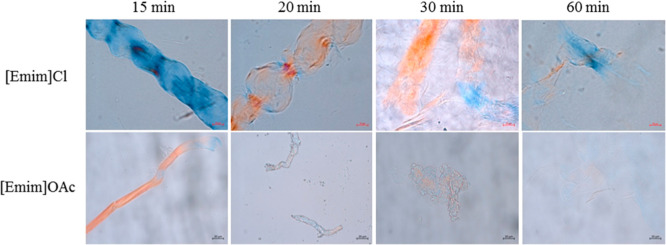
Optical microscopy images (50×) of the cotton fibers in [Emim]­Cl and [Emim]­OAc at different times of dissolution (scale bar 20 μm).

In addition to the dissolution time, Figure 1 also shows distinctions in the dissolution mode of the ILs. The dissolution of cellulose in cotton fibers can occur by five modes of interactions: rapid dissolution by disintegration into fragments (mode 1), large heterogeneous swelling and complete dissolution (mode 2), large heterogeneous swelling and incomplete dissolution (mode 3), homogeneous swelling without dissolution (mode 4), and absence of swelling and dissolution (mode 5).^26^ These modes indicate the quality of the solvents, with quality decreasing from mode 1 to mode 5.

According to Figure 1, dissolution in [Emim]Cl presents a swelling of the fibers, which preceded the dissolution, characterizing the dissolution mode 2. At 20 min, the swelling is not homogeneous, and some zones along the fiber increase their size with the appearance of “balloons”. At 30 min, these zones are no longer observed, suggesting that the balloons have reached maximum expansion and burst.

On the other hand, [Emim]­OAc presented fiber fragmentation without pronounced swelling, characteristic of type 1 dissolution mode. The absence of swelling may be attributed to the superior quality of this solvent that rapidly penetrates the amorphous regions, causing the fibers to rupture along their entire length and disintegrate into rod-like cellulose fragments. Dissolution follows shortly thereafter. This behavior corroborates the faster dissolution observed for cellulose in [Emim]­OAc.

### 3.1 Macroscopic and Microscopic Aspects

The regenerated cellulose filaments were obtained from industrial cotton waste, specifically from textile brushing. Brushing is a finishing process that creates a soft and plush texture by passing the fabric through rollers coated with steel bristles or other abrasive surfaces that lift the fibers. During this process, a residue of very short fibers is generated by mechanical abrasion, as shown in Figure 2a. These short fibers have no commercial value but tend to facilitate the dissolution process compared with other cotton structures, such as fabric scraps and knits.

**Figure 2:**
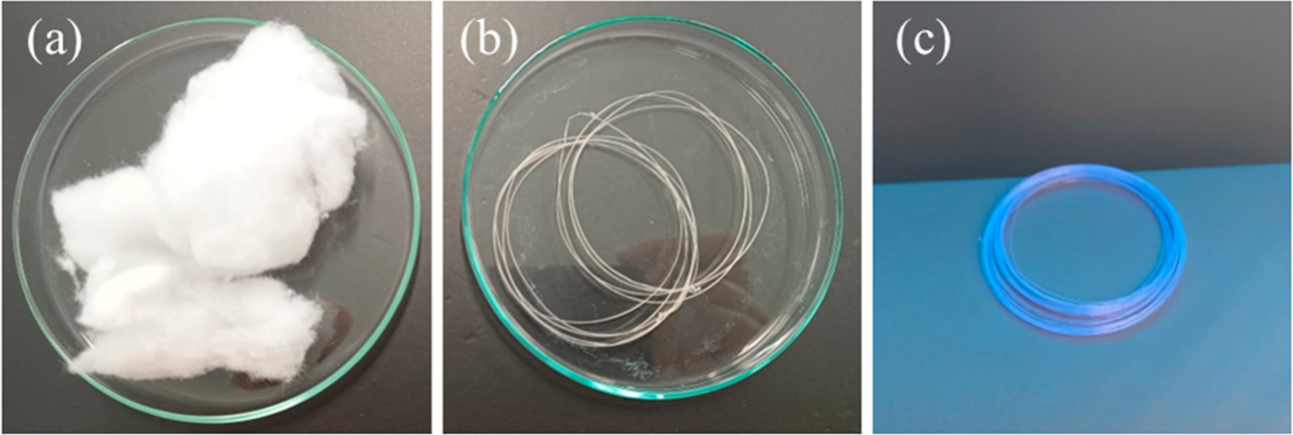
Macroscopic aspect of the cotton waste from the textile brushing process (a), water regenerated cellulose filament (b), and filament under UV-light (c).

Figure 2b shows the macroscopic appearance of the filament obtained by dissolving the residue in the IL [Emim]Cl with subsequent regeneration in water. Macroscopically, there was no difference between the filaments from the different ILs and regenerated in water or ethanol. In general, the filaments were uniform and maintained the color of the residue, including the optical brightener (Figure 2c). Optical brighteners are used to overcome the yellowish shade of chemically bleached textiles, raising the whiteness. These compounds absorb the near-ultraviolet light under solar light and re-emit most of it in the blue range as fluorescence visible under UV light.^27^

The SEM images of the filaments are presented in Figure 3. All of the samples present a homogeneous and compact microstructure, without the evidence of undissolved fibers. The filament’s surface showed grooves and striations, which can be attributed to the double diffusion characteristic of the wet spinning process. The double diffusion is caused by the migration of IL present in spinning dope to the coagulation bath and the migration of coagulant into cellulose dope during fiber solidification, generating internal and external differences in the filament.^28,29^ Consequently, the fiber formation and the regenerated fibers’ properties are influenced by the interactions between the ILs and the antisolvents present in the coagulation bath. A fast counter diffusion caused by a strong coagulant tends to result in more surface irregularities on the fibers. In this scenario, cellulose precipitates in the coagulation bath before the polymer chains have sufficient time to interact, leading to a skin-core layer with a less dense structure. Therefore, one hypothesis for the smoother microstructure of filaments obtained from dissolution in [Emim]­OAc is a slower regeneration rate in the coagulation bath.

**Figure 3:**
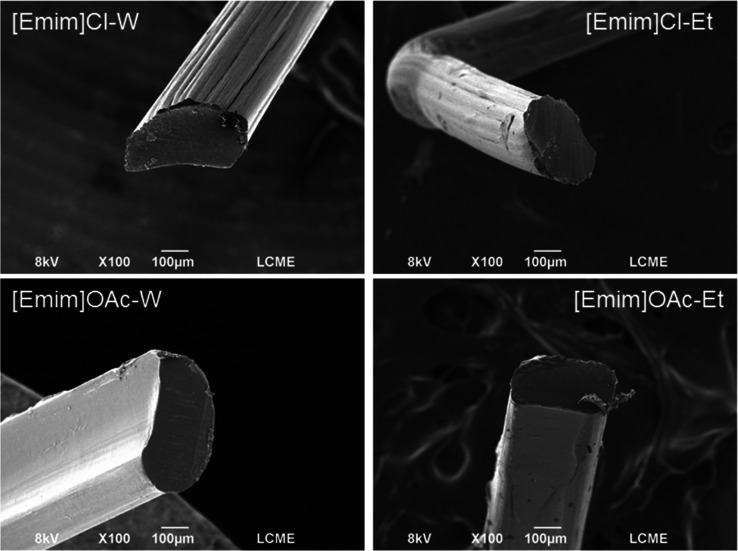
SEM images of the filaments obtained from the dissolution of cellulose in [Emim]­OAc or [Emim]Cl and regeneration in water (W) or ethanol (Et).

### 3.2 Degree of Polymerization

The effect of the dissolution process using both ILs and the composition of the coagulation bath was evaluated on the degree of polymerization (DP). The results are presented in Figure 4 and indicate a significant (*p* < 0.05) reduction in the DP of the regenerated filaments compared to the cotton residue sample. This reduction is expected, as the IL facilitates the breaking of hydrogen bonds and the degradation of smaller molecular chains. During the regeneration process, molecular chains are rearranged into smaller and amorphous structures, resulting in a reduced DP.^30,31^

**Figure 4:**
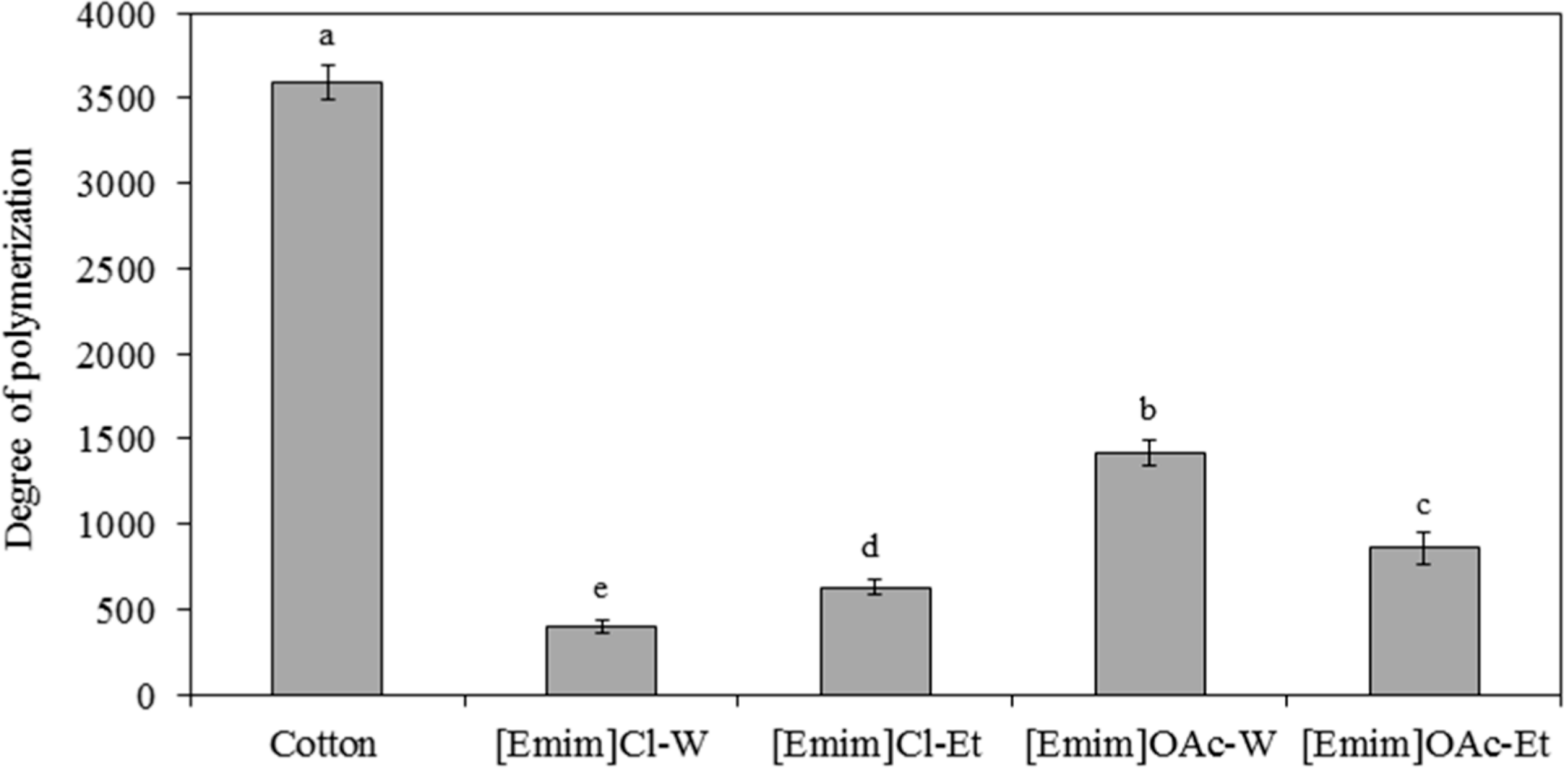
Degree of polymerization of the cotton waste and the filaments obtained from the dissolution of cellulose in [Emim]­OAc or [Emim]­Cl and regeneration in water (W) or ethanol (Et).^a,b^ Columns with the same letter do not differ significantly at *p* < 0.05 according to Tukey’s test.

The filaments obtained from the dissolution of cotton waste in [Emim]­OAc showed a higher DP, indicating that this IL was efficient in breaking hydrogen bonds of cellulose without the degradation of its chains. Regarding the coagulation bath, the behavior of the DP was also different for the two ILs. For [Emim]­OAc, coagulation in water resulted in significantly higher degrees of polymerization than that in ethanol. Water tends to be more effective at disrupting the bonds of the cellulose with the anion of IL due to its greater polarity compared to ethanol.^32^ Gupta et al.^33^ studied the role of water, ethanol, and acetone as antisolvents of cellulose regeneration from dissolution in 1-*n*-butyl-3-methylimidazolium acetate, observing that complete regeneration is achieved in water and a partial regeneration occurs in ethanol.

For filaments obtained from dissolution in [Emim]­Cl, the highest DP was obtained in ethanol, indicating the establishment of a more complete regeneration. A correlation with the microscopy images (Figure 3) indicates that the filament [Emim]­Cl-Et presents a denser and less striated microstructure on the surface than the filament [Emim]­Cl-W. According to Shen et al.,^29^ the water used as a coagulating agent may result in a fast double diffusion process, leading to a skin-core layer with less solidified inner.

### 3.3 XRD

The diffractograms of the cotton waste and the regenerated filaments in water or ethanol after dissolution in [Emim]Cl and [Emim]­OAc are shown in Figure 5.

**Figure 5:**
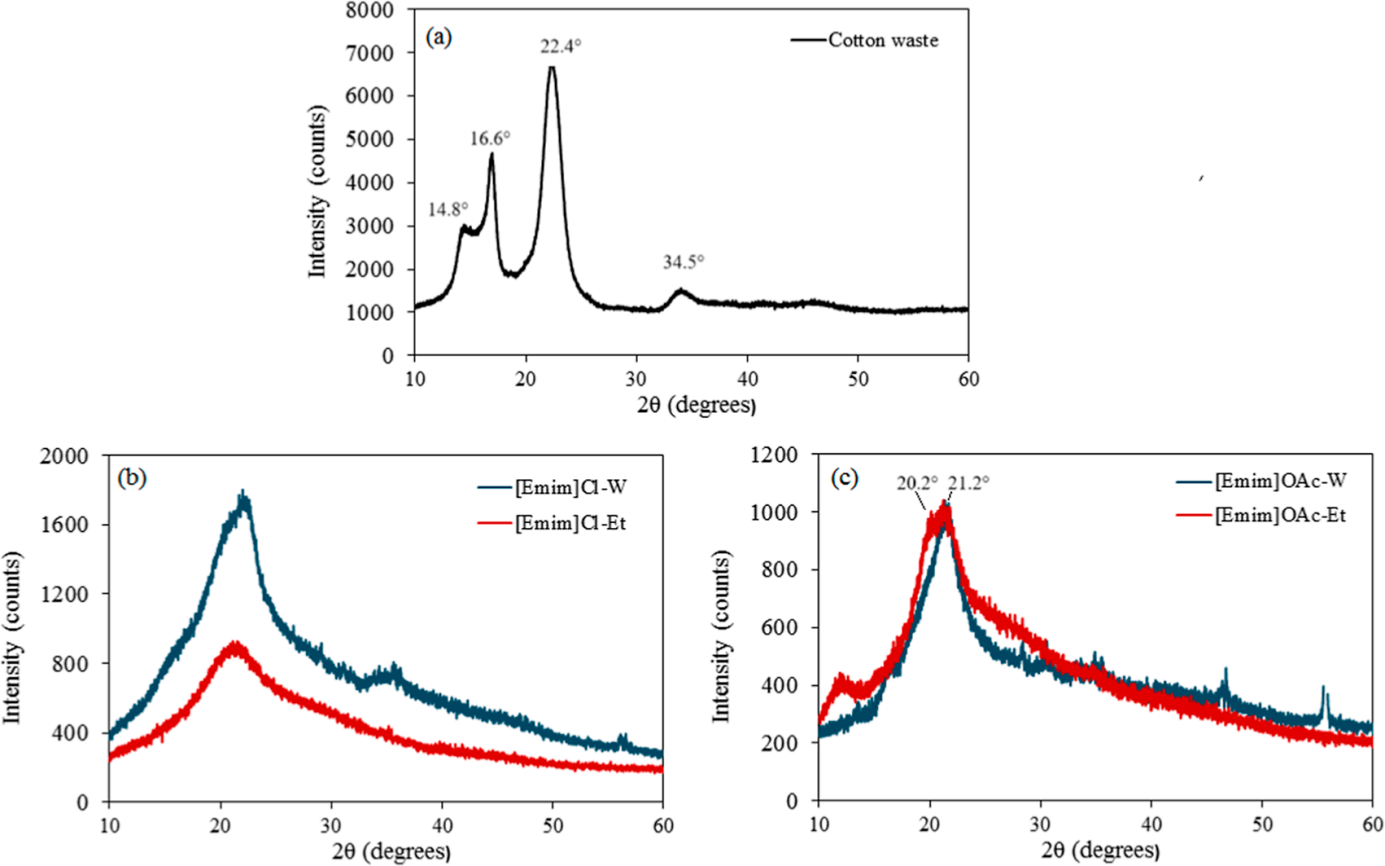
Degree of polymerization of the cotton waste (a) and the filaments obtained from the dissolution of cellulose in [Emim]Cl (b) or [Emim]­OAc (c) and regeneration in water (W) or ethanol (Et).

The cotton waste (Figure 5a) presented typical diffraction angles of cellulose I (2θ) in approximately 14.8°, 16.6°, 22.4°, and 34.5°, corresponding to the (1–10), (110), (200), and (004) crystallographic planes, respectively.^32,34,35^ The peak at 16.6° in pure cellulose is not usually as sharp as that observed in the diffractogram of the residue. This effect may be related to the presence of an optical brightener. The CrI calculated by Segal’s method was 69.6%, which agrees with the CrI of 68.29% obtained for cotton clothes.^36^

The diffractograms of the regenerated filaments from the dissolution of cellulose in [Emim]Cl for both antisolvents exhibited different XRD patterns, with an amorphous structure.^37–39^ The difficulty in recrystallization may be due to the rapid regeneration process, which freezes the cellulose molecules in a disordered state.^39^

For filaments from the dissolution of cellulose in [Emim]­OAc, differences were observed regarding the antisolvents. The water coagulation bath resulted in an amorphous structure, while ethanol exhibited diffraction peaks at 2θ = 20.2° (110), 21.2° (020), and a small broad peak at 2θ = 12.8° (−110), indicating the rearrangement of the hydrogen-bonding network in the stable cellulose II crystalline structure.^40^

These differences may be attributed to variations in the rate of double diffusion during cellulose regeneration, with ethanol exhibiting a lower diffusivity. Fan et al.^41^ suggest that the crystallinity of regenerated cellulose has an inverse relationship with the molecular diffusion rates of antisolvent molecules. These authors observed that the regeneration of cellulose after dissolution of 1-butyl-3-methylimidazolium chloride ([Bmim]­Cl) resulted in an amorphous structure for regeneration in water and a more crystalline structure for regeneration in ethanol, which has a significantly lower diffusivity.

This study indicates that the IL used in cellulose dissolution may also influence antisolvent diffusion since both regenerated filaments from dissolution in [Emim]Cl presented an amorphous structure.

### 3.4 FTIR

The FTIR spectra of the samples of cotton waste and the different filaments are presented in Figure 6. Cotton waste spectra presented characteristic bands of cellulose I. The bands at 3287 and 3333 cm^–1^ correspond to the stretching of the hydroxyl groups (OH) and are attributed to the hydrogen bonding of cellulose I. The band at 2925 cm^–1^ is attributed to the CH stretching vibration. The band at 1630 cm^–1^ is due to OH groups.^35^ Absorption at 1430 cm^–1^ is characteristic of the deformation of the CH_2_ groups. The band at around 1056 cm^–1^ is related to the skeletal vibration of the C–O–C anhydroglucose ring.^42^ The strong band at around 1020 cm^–1^ is attributed to the characteristic C–O–O elongation.

**Figure 6:**
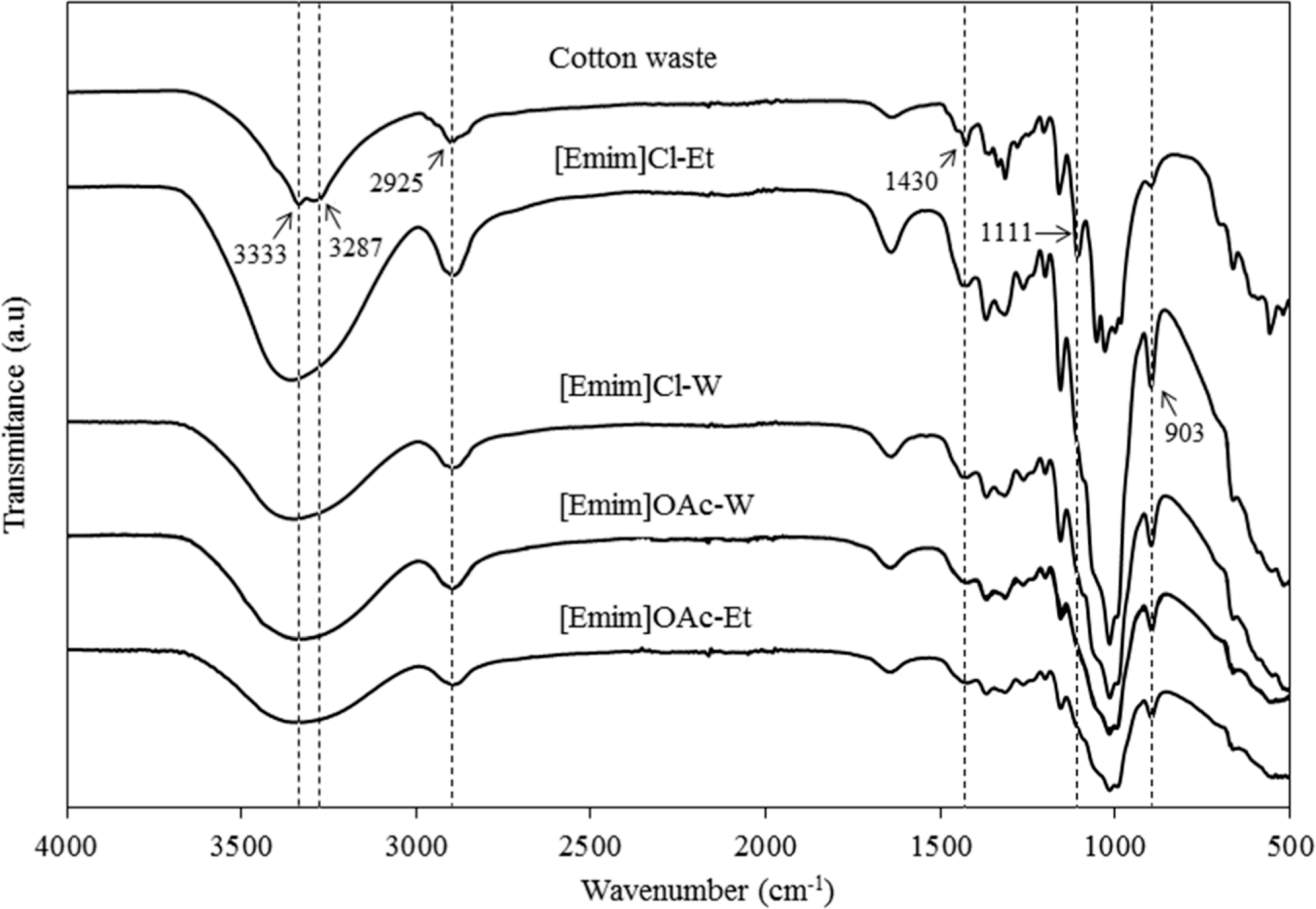
FTIR spectra of the cotton waste and the filaments obtained from the dissolution of cellulose in [Emim]­OAc or [Emim]Cl and regeneration in water (W) or ethanol (Et).

The spectra of the regenerated filaments showed differences (marked with the dotted line) compared with the cotton waste. The two vibrations in the region 3200–3600 cm^–1^ become a broad vibration.^43^ The band of cellulose I at 1430 cm^–1^ was shifted to a broad band at 1420 cm^–1^, indicating the destruction of an intramolecular hydrogen bond involving O at C6.^44^ The band at 1111 cm^–1^ was not present in regenerated filaments, confirming crystal transformation from cellulose I to cellulose II and amorphous cellulose.^35^

In the region of 900 cm^–1^, bands of greater intensity were observed for the regenerated filaments rather than for the cotton residue. This band represents the amorphous region of the cellulose, attributed to the β bond of cellulose.^45^ This change indicates that the processes of dissolution and regeneration result in more amorphous structures. Among the different samples, the band was more intense for samples resulting from dissolution in [Emim]­Cl, suggesting a more amorphous structure.

Regarding the different ILs and coagulants, the structure of cellulose remained unchanged, with no reactions occurring other than the breaking of hydrogen bonds during the dissolution and regeneration processes. These results confirm that ILs act as nonderivatizing solvents for cellulose.

### 3.5 TGA

The thermal behavior of the cotton sample and regenerated cellulose filaments was investigated by TGA, and the results of weight loss (TG) and the derivative of weight loss (DTG) are presented in Figure 7A,B, respectively.

**Figure 7:**
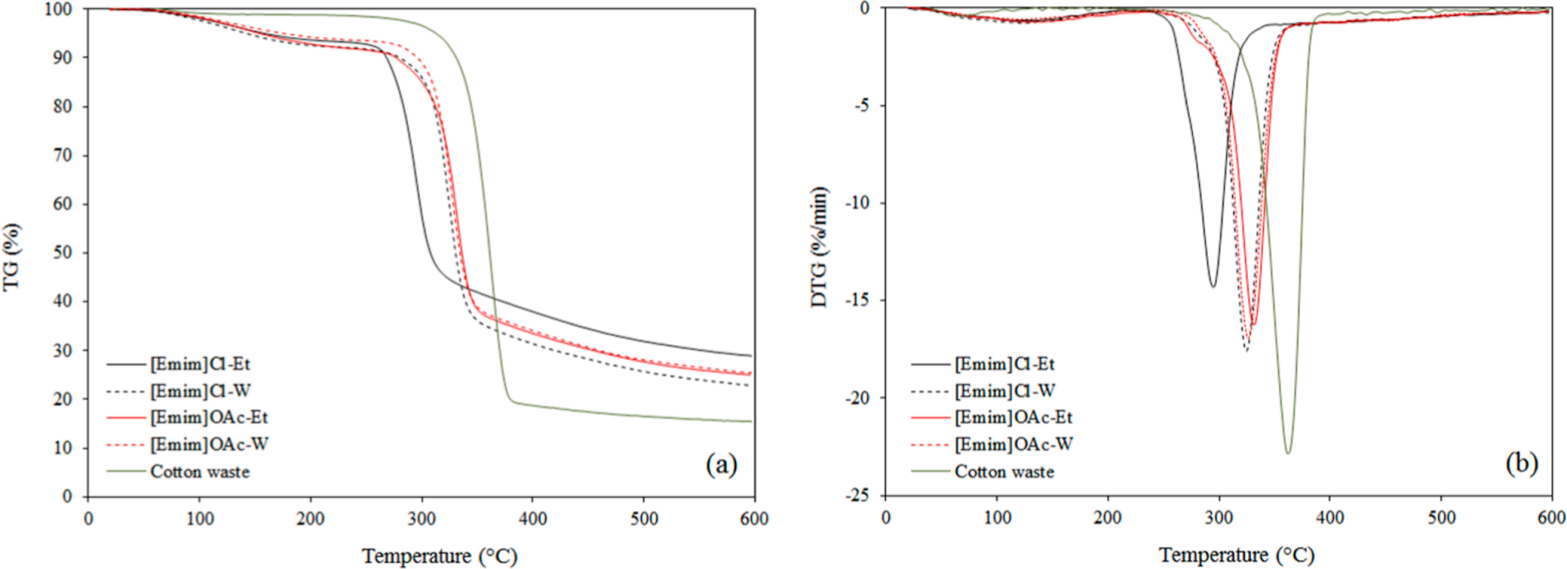
TGA (a) and DTG (b) of the cotton waste and filaments obtained from the dissolution of cellulose in [Emim]­OAc or [Emim]Cl and regeneration in water (W) or ethanol (Et).

The thermograms indicated a small event around 100 °C, attributed to the volatilization of water from the samples.^35^ All samples showed a main degradation step related to cellulose decomposition by Tan et al.,^32^ but differences were observed regarding the temperatures, as summarized in Table 1. The temperature of the maximum decomposition rate (*T*_max_) of the cotton waste was higher than the regenerated filaments, which correlates well with the loss of crystallinity.^46,47^ Reduced crystallinity in cellulose samples leads to lower degradation temperatures due to the formation of a liquid intermediate, reducing activation energy and accelerating dehydration reaction.^48^ Among the different filaments, the lowest thermal stability was observed for [Emim]­Cl-Et, which suggests that this filament has the most amorphous structure.

**Table 1:** Data from DTG of the Cotton Waste and the Filaments Obtained from the Dissolution of Cellulose in [Emim]­OAc or [Emim]Cl and Regeneration in Water (W) or Ethanol (Et)

sample	*T*_onset_ (°C)	*T*_endset_ (°C)	*T*_max_ (°C)	mass loss (%)	char (%)
cotton	244.3	386.0	361.2	79.32	15.47
[Emim]Cl-W	220.3	359.5	322.3	57.36	22.89
[Emim]Cl-Et	214.1	335.9	293.7	50.24	28.98
[Emim]OAc-W	228.8	358.2	325.8	56.13	25.47
[Emim]OAc-Et	225.5	359.7	329.9	55.31	25.05

Results in Table 1 also indicated that the char residue at 600 °C of the regenerated cellulose was higher than that of cotton waste. This increase may also be related to the noncrystalline content, as observed by Liu et al.^49^ The [Emim]­Cl-Et filament presented the highest char residue value, which is consistent with the lowest Tmax.

### 3.6 Mechanical Properties

The mechanical properties of regenerated cellulose filaments in water and ethanol are shown in Table 2.

**Table 2:** Mechanical Properties of the Cellulose Filaments Obtained from the Dissolution of Cellulose in [Emim]­OAc or [Emim]Cl and Regeneration in Water (W) or Ethanol (Et)^a^

sample	tensile strength (MPa)	tenacity (cN/Tex)	elongation (%)	Young’s modulus (GPa)
[Emim]Cl-W	147.78 ± 26.76^ab^	9.01 ± 0.63^a^	6.95 ± 0.89^a^	13.25 ± 4.26^a^
[Emim]Cl-Et	122.78 ± 33.89^b^	5.98 ± 0.41^b^	4.85 ± 0.75^a^	9.88 ± 1.93^b^
[Emim]OAc-W	159.72 ± 35.33^a^	9.33 ± 0.52^a^	6.03 ± 0.98^a^	10.44 ± 1.50^ab^
[Emim]OAc-Et	172.09 ± 23.57^a^	10.06 ± 0.74^a^	5.60 ± 0.99^a^	10.01 ± 1.65^ab^

a Results are presented as mean ± standard deviation. ^a,b^Means followed by the same letter in the same columns do not differ significantly at *p* < 0.05 according to Tukey’s test.

Filaments obtained from the dissolution in [Emim]­Cl and regenerated in ethanol tend to exhibit lower TS, tenacity, and Young’s modulus than other samples. These filaments also presented a lower average linear density (95.3 Tex) than did the filaments obtained from dissolution in [Emim]­OAc (127.9 Tex) without significant differences between the antisolvents. The inferior mechanical resistance may be related to the low DP and the more amorphous structure. According to Pang et al.,^35^ the mechanical properties of cellulose are likely determined by the synergistic effect of crystallinity and DP value.

Ma et al.^50^ investigated the properties of cellulose fibers regenerated from dissolution in 1-butyl-3-methylimidazolium acetate and DMSO using different waste cellulose. They observed tenacity ranging from 8.4 to 22.5 cN/Tex, a modulus from 6.6 to 20.3 cN/Tex, and an elongation from 3.8 to 9.9 cN/Tex. De Silva and Byrne^45^ obtained cellulose fibers using 1-allyl-3-methylimidazolium chloride and regeneration in water and observed a linear relation of the TS with DP. The TS values ranged from 57.5 MPa (495 DP) to 186.5 MPa (2680 DP). In general, the results obtained are consistent with those usually observed in processes with ILs, with TS of 30–375 MPa and a tenacity of 9–25 cN/tex.^51^ Traditional regenerated fibers present a similar modulus of elasticity to those obtained, such as viscose (10.8 GPa) and Modal (13.2 GPa), but higher TS values, with 340 MPa for viscose and 437 MPa for modal.^52^

It is also important to highlight that some studies involving regenerated cellulose fibers and ILs have already shown outstanding mechanical properties. Ioncell technology, for instance, employs a superbase–based IL—1,5-diazabicyclo[4.3.0]­non-5-enium acetate ([DBNH]­[OAc]) to dissolve approximately 15% of pulp. Fibers with tenacity exceeding 50 cN/tex and Young’s modulus around 30 GPa can be obtained through dry–jet wet spinning.^53,54^ Young’s modulus around 40 GPa was also obtained for dry-jet wet-spun fibers using 1-ethyl-3-methylimidazolium diethyl phosphate/DMSO solutions with around 20% cellulose.^55^

However, it should be considered that the cellulose concentration in solution was relatively low (3 wt %) in this study, and the resulting fibers had a relatively large diameter. In thinner fibers, mechanical stresses are typically distributed more uniformly across the cross-section. Therefore, significant improvements are expected with further research involving higher cellulose contents and smaller spinneret diameters.

## 4 Conclusion

Cotton textile waste has shown promise as a raw material for producing regenerated cellulose filaments with imidazolium ILs [Emim]Cl and [Emim]­OAc. The IL and the coagulating agent influence the filaments’ DP, crystallinity, and thermal properties. [Emim]­OAc promoted a faster dissolution and lower depolymerization. The effect of ethanol as a coagulant was dependent on the interaction with an IL: filaments from [Emim]­Cl-ethanol systems resulted in an amorphous structure with lower thermal stability and mechanical properties, whereas filaments from [Emim]­OAc-ethanol maintained a crystalline structure of type II cellulose, with better thermal stability and higher mechanical properties. Water resulted in similar properties for both ILs. Notably, the optical brightener present in the cotton waste remained in the filament. This feature, combined with the potential for improved mechanical properties through a higher cellulose content, suggests positive prospects for the circularity, economy, and sustainability of the textile sector.
